# Protective Role of Rabbit Nucleotide-Binding Oligomerization Domain-2 (NOD2)-Mediated Signaling Pathway in Resistance to Enterohemorrhagic *Escherichia coli* Infection

**DOI:** 10.3389/fcimb.2018.00220

**Published:** 2018-06-26

**Authors:** Mengjiao Guo, Rong Li, Qianqian Xiao, Xiuxiu Fan, Ning Li, Yingli Shang, Liangmeng Wei, Tongjie Chai

**Affiliations:** ^1^Shandong Provincial Key Laboratory of Animal Biotechnology and Disease Control and Prevention, Sino-German Cooperative Research Centre for Zoonosis of Animal Origin of Shandong Province, Shandong Provincial Engineering Technology Research Center of Animal Disease Control and Prevention, College of Animal Science and Veterinary Medicine, Shandong Agricultural University, Tai'an, China; ^2^Collaborative Innovation Center for the Origin and Control of Emerging Infectious Diseases, Taishan Medical University, Tai'an, China

**Keywords:** rabbits, NOD2, NF-κB, antibacterial ability, EHEC

## Abstract

Nucleotide-binding oligomerization domain 2 (NOD2), a member of the NOD-like receptors (NLRs) family that is well-known to play a key role in innate immune responses and is involved in innate antibacterial responses. In this study, rabbit NOD2 (rNOD2) was cloned from rabbit kidney (RK) cells. It was distributed in various tissues, and the highest level of *rNod2* was detected in spleen. Moreover, the expression of *rNod2* was significantly upregulated in the heart, liver, and spleen induced by enterohemorrhagic *Escherichia coli* (EHEC). Overexpression of rNOD2 induced the expression of pro-inflammatory cytokine, including *Il1*β, *Il6, Ifn-*γ, and *Tnf*, as well as defensins, including *Defb124, Defb125*, and *Defb128* through the nuclear factor (NF)-κB signaling pathway. Furthermore, overexpression of rNOD2 inhibited the growth of EHEC, and knockdown of rNOD2 or inhibition of the NF-κB pathway promoted its replication. In addition, our results suggest that rNOD2 can significantly activate NF-κB signaling and trigger antibacterial defenses to increase the expression of pro-inflammatory cytokine and defensins after stimulation by EHEC. These findings are useful to further understanding the innate immune system of rabbits and providing a new perspective for the prevention of bacterial diseases in rabbits.

## Introduction

The innate immune system plays a crucial role in the non-specific immune response, and is essential for triggering the acquired immune response against microbial pathogens. The typically conserved exogenous microbial components termed pathogen-associated molecular patterns (PAMPs) or damage-associated molecular patterns are recognized by host germline-encoded pathogen recognition receptors, which can be grouped into four families: nucleotide-binding oligomerization domain (NOD)-like receptors (NLRs), toll-like receptors, retinoid acid-inducible gene-1-like receptors and C-type lectin receptors (CLRs) (Schroder and Tschopp, 2010; Moltke et al., 2013; Lamkanfi and Dixit, 2014). NLRs possess neither signal peptides nor transmembrane domains, and they detect PAMPs that reach the cytosol and trigger innate inflammatory responses and antibacterial immune responses (Elinav et al., 2011). Toll-like receptors have been extensively studied and survey the extracellular environment for microbial pathogens at the cell surface and the luminal side of intracellular vesicles (Kimbrell and Beutler, 2001; Uematsu and Akira, 2006). However, retinoid acid-inducible gene-1-like receptors are intracellular cytosolic sensors and sense viruses (Kawai and Akira, 2006). Compared with other PRRs, CLRs are especially important for antigen-presenting cells. According to molecular structure, they can be classified into soluble and transmembrane CLRs. They not only recognize and take up various antigens, but also transmit signals in cells, helping macrophages and dendritic cells to effectively induce innate immunity (Lepenies et al., 2013; Jung et al., 2018).

During host-microbe interactions, NLRs play a fundamental role in recognizing and responding to PAMPs (Meylan et al., 2006). The NLRs, best characterized in mammals, are multi-domain proteins containing three distinct domains: a C-terminal leucine-rich repeats domain, a central nucleotide-binding domain, and an N-terminal protein-protein interaction domain such as baculovirus inhibitor repeats, caspase recruitment domain (CARD), and pyrin domain (Inohara and Nuñez, 2003). The NOD2 gene belongs to the subfamily of NLRs and leads to the induction of multiple effector signaling pathways against pathogenic microorganisms. The best characteristic of these signaling pathways is to activate nuclear factor (NF)-κB by interacting with receptor-interacting protein 2 (Hasegawa et al., 2008). Consequently, NOD2 stimulation actives proinflammatory transcription factors NF-κB, resulting in the expression of cytokine (Gutierrez et al., 2002; Correa et al., 2012), as well as antimicrobial peptides, which involved in the antimicrobial response (Voss et al., 2006). Specifically, the reduction of NF-κB and related impairment of cell adhesion molecule expression were observed in NOD2^−/−^ mice challenged with *Escherichia coli* (*E. coli*). Moreover, an increase in bacterial colonization and a reduction in neutrophil, cytokine, and chemokine were also detected (Theivanthiran et al., 2012).

Muramyl dipeptide (MDP), which is produced by all bacteria (Girardin et al., 2003b; Inohara et al., 2003), is the essential bacterial component which can be recognized by NOD2 (Nakamura et al., 2014). At present, the enzymes necessary for the production of MDP are found only in bacteria (Girardin et al., 2003a; Lenz et al., 2003), indicated that NOD2 can resist various bacterial pathogens. In addition, NOD2 has been shown to respond to peptidoglycan from bacterial extracts, gram-negative bacteria, and gram-positive bacteria (Hasegawa et al., 2006). NOD2 has been reported that it can inhibit the growth of a variety of specific bacteria, for instance, *Shigella flexneri*, and *Salmonella typhimurium* (Hisamatsu et al., 2003; Kufer et al., 2006).

Rabbits have been increasingly utilized as a food source and as popular pet in households. The rabbit immune system has attracted more and more attention because of its potential as an experimental model for human disease characterized by a lower cost (Cantey and Blake, 1977). However, diarrhea and rapid death caused by enterohemorrhagic *Escherichia coli* (EHEC) in weaned rabbits results in large losses in rabbit breeding. To date, the rabbit immune system and the relationship between it and pathogen recognition receptors are rarely studied. Although the crystal structure of rabbit NOD2 (rNOD2) has been studied (Sakiko et al., 2016), the rNOD2-mediated signaling pathway and antimicrobial activity remain unknown. Given this situation, rNOD2 was cloned and characterized from rabbit kidney (RK-13) cells. Moreover, the expression of rNOD2 in various tissues from healthy rabbits were detected. The main signaling pathway and antibacterial activity of rNOD2 were also investigated. Our study clarifies the role of rNOD2 in NF-κB activation and showed that the triggering of antibacterial defenses is conserved during EHEC infections in rabbits.

## Materials and methods

### Reagents, bacteria, cells, and animals

BAY11-7082 (an NF-κB inhibitor) was obtained from MedChem Express (Monmouth Junction, NJ, USA). MDP was obtained from InvivoGen (San Diego, CA, USA).

The EHEC used in this study was isolated from clinically acute diarrhea rabbits and grown in a nutrient broth medium at 37°C for 12 h.

RK-13 cells were cultured in Dulbecco's modified Eagle medium (DMEM; GIBCO, Grand Island, NY, USA) supplemented with 10% fetal bovine serum (Transgen, Beijing, China) at 37°C and 5% (v/v) CO_2_.

Thirty-five-day-old healthy New Zealand White rabbits were purchased from a farm, and given sufficient water and feed. All animal experiments were handled in accordance with the appropriate biosecurity guidelines. This study was carried out in accordance with the recommendations of the Shandong Agricultural University Animal Care and Use Committee (no. SDAUA-2015-005).

### Cloning and analysis of the rNOD2 sequence

Total RNA was extracted from the RK-13 cells with the *Trans*Zol up (Transgen) and cDNA synthesis was carried out with TransScriptR One-step gDNA Removal and cDNA Synthesis SuperMix (Transgen). To obtain the sequence of rNOD2, degenerate primers were designed based on the predicated gene (NCBI XM_008261590.2) (Table 1). A phylogenetic tree of rNOD2 was made using the neighbor joining method of the MEGA5.1 program.

**Table 1:** Primers used in this study.

**Primer name**	**Sequence(5′-3′)**	**Purpose**
*rNOD2* F	ggctggcggttgtgaa	Gene cloning
*rNOD2* R	acagagctcagtcggaatgg	
*qdrNOD2* F	caacctcaagggcttctcag	RT-PCR
*qdrNOD2* R	caggaaatgctgcaagatca	
*Il1β* F	tggcacgtatgagctgaaag	RT-PCR
*Il1β* R	ggccacaggtatcttgtcgt	
*Il4* F	cactccggcagttctacctc	RT-PCR
*Il4* R	gcagaggttcctgtcgagtc	
*Il6* F	ctgaagacgaccacgatcca	RT-PCR
*Il6* R	aaggacacccgcactccat	
*Il8* F	ctctcttggcaaccttcctg	RT-PCR
*Il8* R	ttgcacagtgaggtccactc	
*Il10* F	aaaagctaaaagccccagga	RT-PCR
*Il10* R	cgggagctgaggtatcagag	
*Ifn-γ* F	ctcgaatttcggtggatgat	RT-PCR
*Ifn-γ* R	agcgtctgactcctttttcg	
*Tnf* F	cacttcagggtgatcggc	RT-PCR
*Tnf* R	tgcgggtttgctactacg	
*Defb124* F	gcaccaagcaagagtccttc	RT-PCR
*Defb124* R	acgccagagccagctactta	
*Defb125* F	cgtgctgcatctccttaaca	RT-PCR
*Defb125* R	gcgaagcagaaaattgatcc	
*Defb128* F	gggctcaaggctttctcttt	RT-PCR
*Defb128* R	aaatctcgcctagcttgcac	
*Defb135* F	gctgcatctccaaatccaat	RT-PCR
*Defb135* R	tagtgggatggtgcaactga	
*Np5* F	aggcaggcgtgttctgtact	RT-PCR
*Np5* R	ggtctccacgcaaataagga	
*Gapdh* F *Gapdh* R	aggtcatccacgaccacttc gtgagtttcccgttcagctc	RT-PCR

### Expression analysis of rNOD2 *in vivo*

Five healthy rabbits were randomly selected and euthanized, and the spleen, duodenum, jejunum, pancreas, rectum, cecum, esophagus, appendix, trachea, stomach, lymph follicles, mesenteric lymph nodes, sacculus rotundus, colon, ileum, thymus, cerebellum, brainstem, brain, kidney, lung, muscle, heart, liver, and skin were collected to determine tissue distribution of rNOD2 by quantitative real time PCR (qRT-PCR).

The rabbits were intraperitoneally injected with of EHEC bacterial suspension (10^8^ CFU per rabbit). Five rabbits from each group were randomly selected and killed, and the kidney, liver, and spleen were collected at 1, 2, and 3 days post infection (dpi). Primers of *qdrNOD2* F and *qdrNOD2* R were selected for analysis expression of rNOD2 (Table 1).

### Recombinant expression of rNOD2

The rNOD2 was amplified with the primers in Table 1. The fragment was subcloned into a pCDNA3.1 (+) vector using a Hieff Clone™ Multi One Step Cloning Kit (Yeasen, Shanghai, China) to yield the constructs pC-rNOD2. RK-13 cells were plated in 6-well plates for 12 h to transfection. Two micrograms of pC-rNOD2 or pCDNA3.1 (+) vector were transfected with *Trans*IL-LT1 Transfection Reagent (Mirusbio, MI, USA) for 24 h. pCDNA3.1 (+) vector was used as a control.

### Immunofluorescence labeling

Cells were fixed with 4% paraformaldehyde and then permeabilized with 0.1% Triton X-100 for 10 min. After blockade of nonspecific binding with 5% bovine serum albumin in PBS for 30 min, cells were incubated with HA Tag Monoclonal antibody (Abbkine, USA), and then incubated with fluorescein isothiocyanate (FITC)-goat anti-mouse IgG. Images were visualized with a Leica fluorescence microscope.

### Western blotting

Cells were lysed with ice-cold RIPA buffer containing a protease inhibitor (Beyotime). Protein samples were boiled in SDS sample buffer, and then run on SDS-PAGE. The separated proteins were transferred to a PVDF membrane. Membrane was blocked with 5% skim milk for 1 h. HA tag monoclonal antibody was used to detected rNOD2. Then Membrane was incubated with secondary antibodies. β-actin was used as control. Protein bands were visualized with a ChemiDoc XRS (Bio-Rad, Marnes-la-Coquette, France) by a Western ECL Substrate kit.

### Small interfering (si) RNA interference

The siRNA sequences (si-rNOD2-1, si-rNOD2-2, and si-rNOD2-3) targeting the rNOD2 and negative control (NC) siRNA were synthesized by company (GenePharma, Shanghai, China). One microgram of siRNA and NC siRNA were transfected with *Trans*IL-LT1 Transfection Reagent. NC siRNA was used as a control. The si-RNA sequences that we used were as follows: si-rNOD2-1, sense 5′-CCUGGACACUGUCUGGAAUTT-3′, antisense 5′-AUUCCAGACAGUGUCCAGGTT-3′; si-rNOD2-2, sense 5′-GCAGGACUUUCAGGAAUUUTT-3′, antisense 5′-AAAUUCCUGAAAGUCCUGCTT-3′; si-rNOD2-3, sense 5′-GCACUGAGUUCAACCUCAATT−3′, antisense 5′-UUGAGGUUGAACUCAGUGCTT−3′;NC, sense 5′-UUCUCCGAACGUGUCACGUTT-3′, antisense 5′-ACGUGACACGUUCGGAGAATT-3′.

### Luciferase assays

The RK-13 cells were cultured in 24-well plates and grown in standard conditions for 12 h prior to transfection. The luciferase reporter plasmids (pGL3-NF-κB and pGL3-IFN-β) were purchased from Agilent (Santa Clara, CA, USA). The pRL-TK plasmid (Promega, Madison, WI, USA) acted as an internal control to normalize transfection efficiency. 1 μg of pC-rNOD2 or pCDNA3.1 (+) vector and 500 ng of si-rNOD2 or NC siRNA with 100 ng of reporter plasmid and 50 ng of pRL-TK plasmid were transfected into cells using *Trans*IL-LT1 Transfection Reagent. After 24 h, the cells were lysed and harvested, and luciferase activities were examined by a dual-luciferase reporter assay system (Beyotime, Wuhan, China).

### EHEC infection

The RK-13 cells were cultured in 24-well plates and grown in standard conditions for 12 h prior to transfection. One microgram of pC-rNOD2 or 500 ng of si-rNOD2 was transfected into cells using *Trans*IL-LT1 Transfection Reagent. pCDNA3.1 (+) vector and NC siRNA were used as control, respectively. After 24 h, EHEC (1 × 10^7^ CFU) were incubated with RK-13 cells for 2 h. Then, the bacteria were removed and washed 3 times with PBS containing 100 μg/ml gentamicin. The RK-13 cells were cultured in DMEM containing 100 μg/ml gentamicin for 3 h. Then RK-13 cells were lysed in 1% Triton X-100. After 20 min, cell lysates were plated onto nutrient agar for intracellular CFU determination. Other RK-13 cells were harvested for RNA extraction.

### qRT-PCR

Total RNA was extracted from the tissues of rabbits and RK-13 cells and reversed as described above. qRT-PCR was carried out using the 7500 Fast Real-Time PCR System (Applied Biosystems, Carlsbad, CA, USA) with TransStartR Tip Green qPCR SuperMix (Transgen Biotech). The primers of *Tnf* and *Il6* were as previously reported (Liu et al., 2017). Other primers were designed using the Primer3 (http://bioinfo.ut.ee/primer3-0.4.0/) based on published target sequences (Table 1). The qRT-PCR conditions consisted of initial denaturation at 94°C for 30 s followed by 40 cycles at 94, and 60°C/34 s. The relative gene expressions were calculated based on the 2^−ΔΔCt^ method using the glyceraldehyde-3-phosphate-dehydrogenase (*Gapdh*) as an endogenous reference gene.

### Statistical analysis

Data represent the means ± standard deviations. Statistical analyses were performed by SPSS 19.0 software (SPSS Inc., Chicago, IL, USA), the significant difference between control and the treated group were evaluated by non-parametric Mann-Whitney U test. We adopted a level of statistical significance of *P* < 0.05.

## Results

### Sequence analysis of rNOD2

The full-length sequence of rNOD2 was obtained from RK-13 cells. The open reading frame contains 3,042 bp and encodes a protein of 1,013 amino acids. The nucleotide sequence of rNOD2 has been deposited to the GenBank (MF125932). As observed in Table 2, rNOD2 exhibited highest amino acid identity with *Ochotona princeps* (91.7%) and *Macaca nemestrina* (86.5%). *Larimichthys crocea* and *Takifugu rubripes* were at the bottom of the table.

**Table 2:** The amino acid homologies of rNOD2 to other NOD2 proteins.

**Species**	**Amino acid identity (%)**
*Ochotona princeps*	91.7
*Macaca nemestrina*	86.5
*Pan troglodytes*	86.4
*Homo sapiens*	86.4
*Pteropus Alecto*	84.4
*Canis lupus familiaris*	84
*Sus scrofa*	83.3
*Cavia porcellus*	83.3
*Mustela putorius furo*	82.7
*Orcinus orca*	81.6
*Ovis aries*	80.9
*Pteropus vampyrus*	80.8
*Mus musculus*	79.7
*Clupea harengus*	45.8
*Ctenopharyngodon idella*	45.4
*Oncorhynchus mykiss*	45.2
*Danio rerio*	44.8
*Salmo salar*	44.8
*Takifugu rubripes*	42.8
*Larimichthys crocea*	42.6

**Figure 1:**
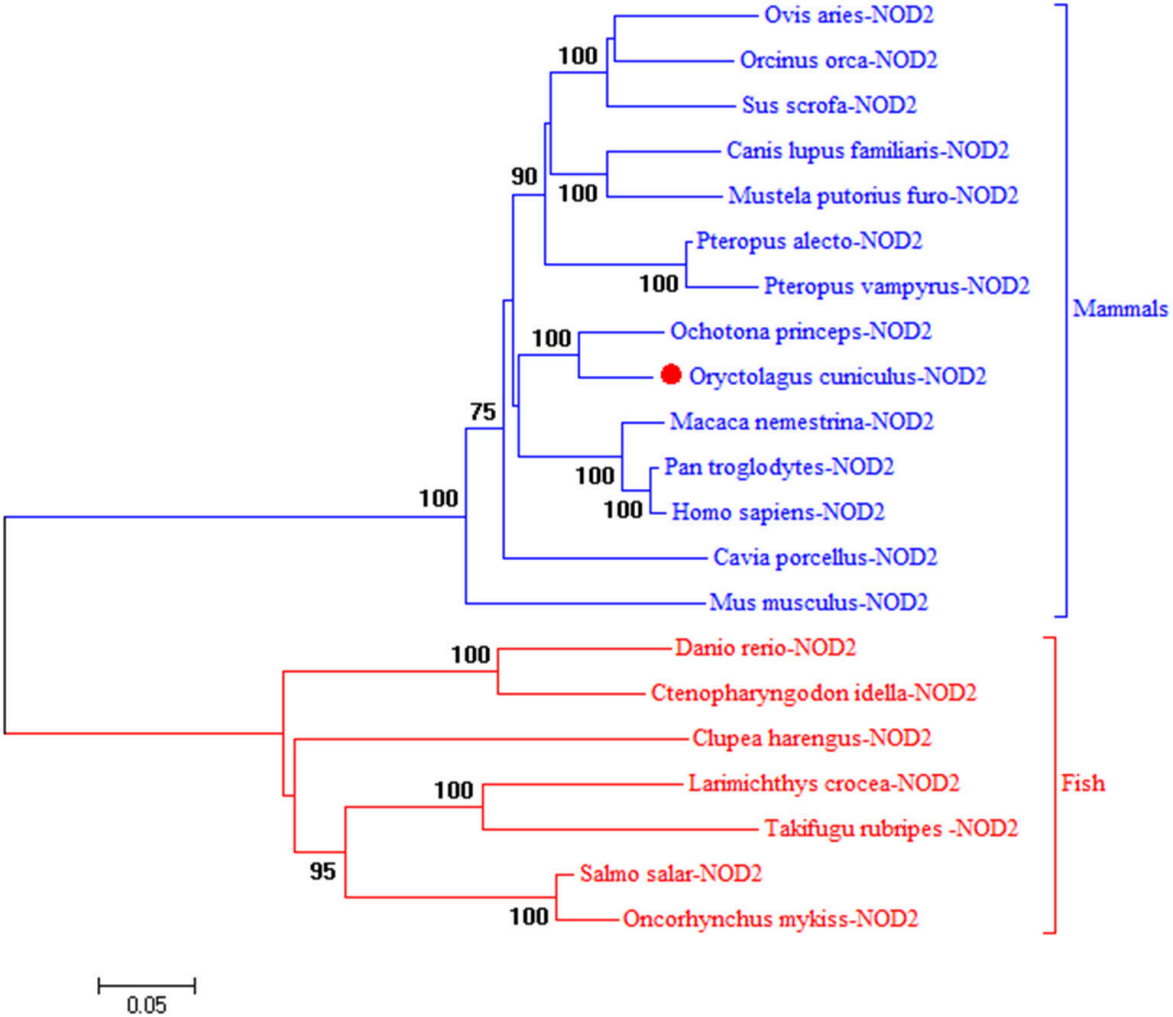
A phylogenic tree based on rNOD2 between *Oryctolagus curiculus* and other species. A neighbor-joining tree was performed with MEGA 5.0. The tree was bootstrapped 1,000 times and the percentage of the bootstrapped values are shown. The sequences used are: *Ovis aries* NOD2, XP_014955920.1; *Orcinus orca* NOD2, XP_004264937.1; *Sus scrofa* NOD2, BAH24204.1; *Canis lupus familiaris* NOD2, NP_001273968.1; *Mustela putorius furo* NOD 2, XP_004744216.1; *Pteropus Alecto* NOD2, XP_006908812.1; *Pteropus vampyrus* NOD2, XP_011358488.1; *O. princeps* NOD2, XP_004584173.1; *M. nemestrina* NOD2, XP_011757602.1; *P. troglodytes* NOD2, NP_001098710.2; *H. sapiens* NOD2, NP_071445.1; *Cavia porcellus* NOD2, XP_003477732.2; *Mus musculus* NOD2, AAN84594.1; *Danio rerio* NOD2, NP_001314973.1; *Ctenopharyngodon idella* NOD2, ACX71753.1; *Clupea harengus* NOD2, XP_012682426.1; *Larimichthys crocea* NOD2, AJF23836.1; *Takifugu rubripes* NOD2, NP_001035913.1; *Salmo salar* NOD2, XP_014031576.1; *Oncorhynchus mykiss* NOD2, NP_001188484.1.

To understand the evolution of rNOD2, a phylogenetic tree of NOD2 was constructed using the amino acids sequences of various species. Because there is no NOD2 in birds, two major branches were observed in Figure 1. As was expected, rNOD2 branched with *O. princeps* and exhibited a stronger evolutionary relation than others among mammalian species.

### Expression pattern of rNOD2 *in vivo*

**Figure 2:**
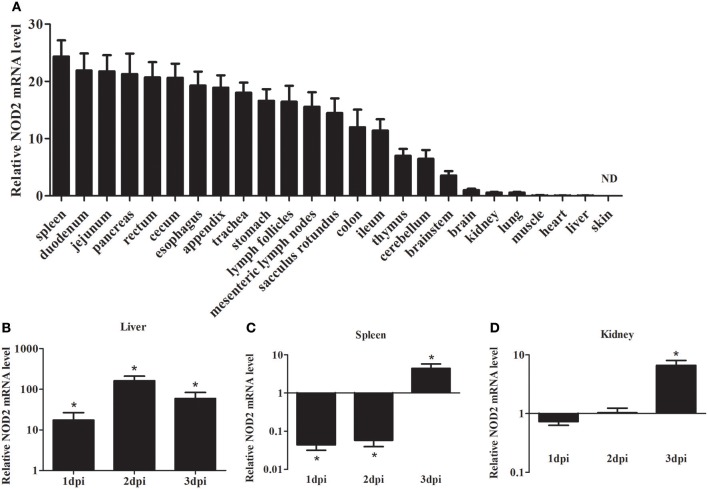
Expression analysis of rNOD2 in rabbits. **(A)** The tissue distribution of rNOD2 transcripts in healthy rabbits. The relative rNOD2 mRNA level in the liver **(B)**, spleen **(C)**, and kidneys **(D)** in rabbits in the early stages of an EHEC (10^8^ CFU) infection. Bars represent the means ± standard deviations (*n* = 5). A significant difference is indicated with an asterisk (*). ND, not detected in skin using qRT-PCR.

As shown in Figure 2A, the expression of *rNod2* was detected in all test tissues, except skin. *rNod2* was strongly expressed in spleen, duodenum, jejunum, pancreas, rectum, and cecum, with the highest level in spleen. The lower ecpression of *rNod2* was detected in the muscle, heart, and liver.

To determine the effect of bacterial infection on *rNod2* expression, rabbits were infected with EHEC. *rNod2* was significantly induced by EHEC, with 17.4-fold (*P* < 0.05) of expression 1 dpi in the liver, and peaked at 2 dpi (162.0-fold, *P* < 0.05) (Figure 2). On the contrary, in the spleen, *rNod2* was significantly downregulated at 1 and 2 dpi and increased by 4.5-fold (*P* < 0.05) at 3 dpi (Figure 2C). The expression of *rNod2* was significantly up-regulated by 6.63-fold (*P* < 0.05) at 3 dpi in the kidney (Figure 2D).

### The expression of rNOD2 in RK-13 cells

**Figure 3:**
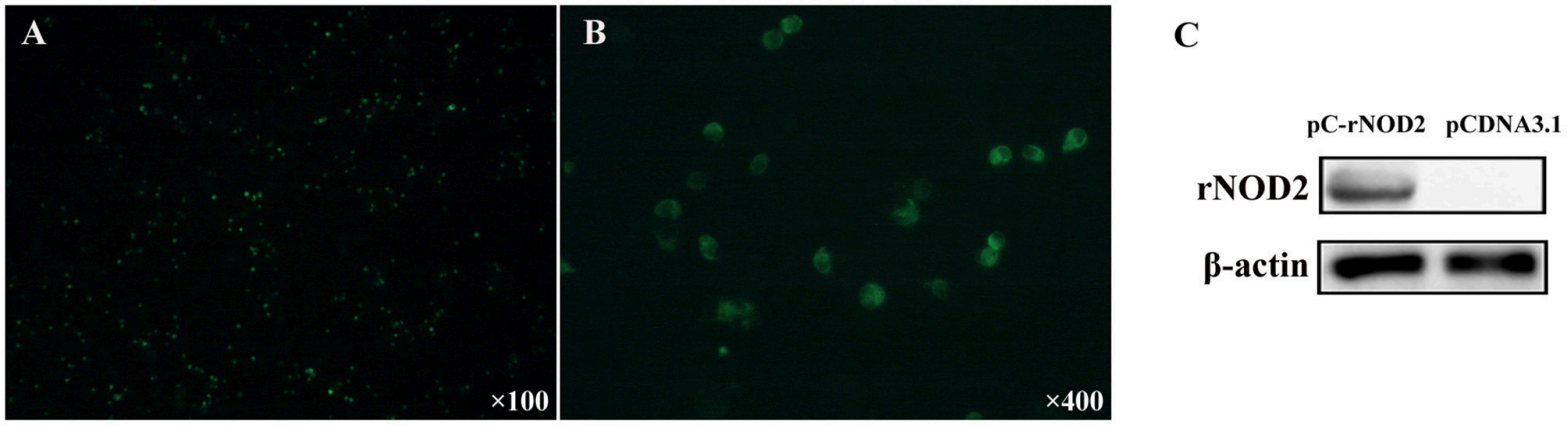
rNOD2 recombinant expression at protein level. The expression of rNOD2 protein in RK-13 cells was detected with immunofluorescence labeling. rNOD2 appears in green. Magnification was 100 × **(A)** and 400 × **(B)**. **(C)** Immunoblotting of rNOD2 after transfected with pC-rNOD2. pCDNA3.1 (+) vector was used as control.

To elucidate the expression of rNOD2 in cells, we constructed pC-rNOD2-expressing RK-13 cells. As shown in Figures 3A,B, rNOD2 was expressed in the intracytoplasm. The result of western blot confirmed that rNOD2 was expressed in high level at protein level (Figure 3C).

### rNOD2 signaling through the NF-κB pathway

**Figure 4:**
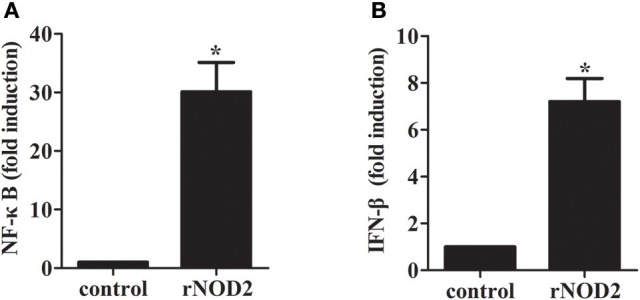
Effect of NF-κB and IFN-β by rNOD2. pC-rNOD2, pCDNA3.1 (+) vector, NF-κB **(A)**, the IFN-β **(B)** reporter plasmid, and pRL-TK plasmid were transfected into cells for 24 h. The cells were harvested for dual-luciferase assays. Bars represent the means ± standard deviations (*n* = 3). A significant difference is indicated with an asterisk (*).

To determine whether rNOD2 is involved in NF-κB activities, recombinant rNOD2 was expressed in RK-13 cells together with a NF-κB reporter construct. As shown in Figure 4A, rNOD2 could significantly activate NF-κB luciferase activities compared with the controls (30.11-fold, *P* < 0.05) after 24 h transfection. In addition, our results also showed that rNOD2 could drive IFN-β expression (7.20-fold, *P* < 0.05, Figure 4B).

### Induction of cytokine and defensin gene expression by rNOD2 effector domains overexpression

**Figure 5:**
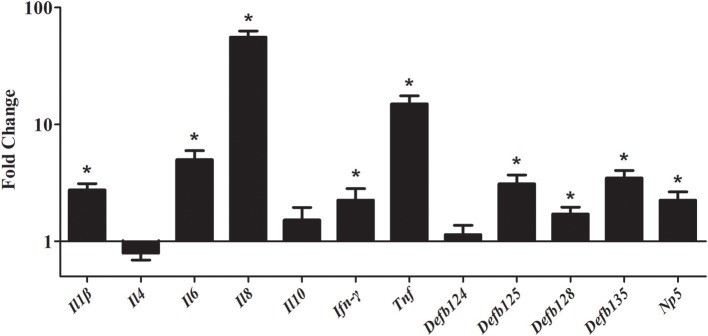
Expression of immune-related genes induced by rNOD2 overexpression in RK-13 cells. Two micrograms of pC-rNOD2 or pCDNA3.1 (+) vector were transfected for 24 h. The expression of immune-related genes induced by overexpression of rNOD2 were determined using RT-PCR. Bars represent the means ± standard deviations (*n* = 3). A significant difference is indicated with an asterisk (*).

To study the gene expression level of cytokines and defensins by rNOD2, RK-13 cells were transfected with pC-rNOD2 or pCDNA3.1 (+) vector. As shown in Figure 5, the pro-inflammatory cytokines, including *Il1*β, *Il6, Il8*, and *Tnf* were significantly elevated by rNOD2 overexpression. In particular, the expression of *Il8* showed highest increase by 55.97-fold (*P* < 0.05; Figure 5). However, anti-inflammatory cytokines, such as *Il4* and *Il10* exhibited no significant difference. The expression of *Ifn-*γ was upregulated 2.25-fold (*P* < 0.05) by rNOD2 overexpression. When the expression of β-defensin *Defb124, Defb125, Defb128, Defb135*, and α-defensin *Np5* was investigated, all of these genes exhibited significant upregulation with the overexpression of rNOD2 except for *Defb124*.

### rNOD2 induces EHEC-stimulated NF-κB

**Figure 6:**
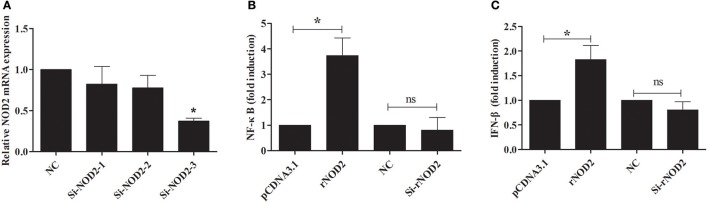
rNOD2 induces EHEC-stimulated NF-κB and IFN-β. **(A)** Silencing efficiency of siRNA targeting rNOD2. RK-13 cells were transfected with the indicated siRNA1, 2, and 3 for 24 h. qRT-PCR was used to analyze the expression of rNOD2. pC-rNOD2, pCDNA3.1 (+) vector, Si-rNOD2, and NC siRNA with NF-κB **(B)** and IFN-β **(C)** reporter plasmid, and pRL-TK plasmid were transfected into RK-13 cells. After 24 h, the cells were infected with EHEC. The cells were then harvested for dual-luciferase assays. Bars represent the means ± standard deviations (*n* = 3). A significant difference is indicated with an asterisk (*).

si-rNOD2-1, si-rNOD2-2, and si-rNOD2-3 were designed to target the different positions of rNOD2. The knockdown efficiency of Si-rNOD2-3 was 37.25% (Figure 6A). rNOD2 increased the induction of EHEC-stimulated NF-κB by 3.74-fold (*P* < 0.05, Figure 6B) and slightly activated IFN-β (*P* < 0.05, Figure 6C). However, rNOD2 knockdown inhibited the EHEC-stimulated induction of NF-κB and IFN-β (*P* > 0.05, Figures 6B,C).

### Induction of cytokine and defensin gene expression by rNOD2 after infection with EHEC

**Figure 7:**
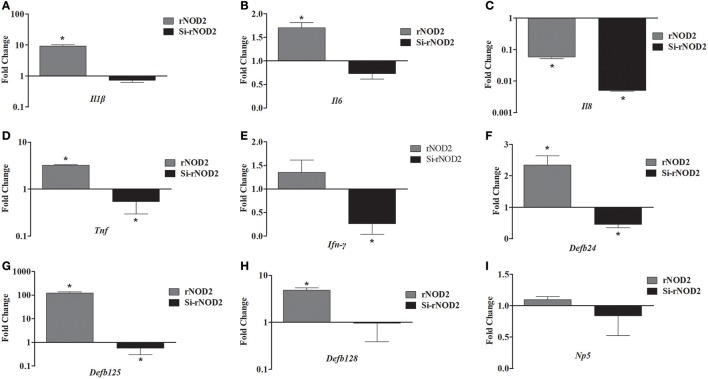
Effect of overexpression or knockdown of rNOD2 on expression of immune-related genes induced by EHEC in RK-13 cells. Expression of *Il1*β **(A)**, *Il6* **(B)**, *Il8* **(C)**, *Tnf* **(D)**, *Ifn-*γ **(E)**, *Defb124* **(F)**, *Defb125* **(G)**, *Defb128* **(H)**, and *Np5* **(I)** in EHEC-infected RK-13 cells transfected with pC-rNOD2 or si-rNOD2. Bars represent the means ± standard deviations (*n* = 3). A significant difference is indicated with an asterisk (*).

The effect of overexpression or knockdown of rNOD2 on cytokines and defensins production induced by EHEC was explored. As shown in Figure 7, an increased expression of pro-inflammatory cytokine, including *Il1*β (Figure 7A), *Il6* (Figure 7B), and *Tnf* (Figure 7D) as well as β-defensin, including *Defb124* (Figure 7F), *Defb125* (Figure 7G), and *Defb128* (Figure 7H) could be observed in cells transfected with rNOD2. For example, the expression of *Il1*β was increased significantly 9.31-fold (*P* < 0.05) by rNOD2 overexpression (Figure 7A). On the contrary, the expression of *Il8* was significantly down-regulated 0.06-fold (*P* < 0.05, Figure 7C), and no obvious changes were observed in *Ifn-*γ by rNOD2 overexpression (Figure 7E). However, knockdown from rNOD2 impaired EHEC-induced pro-inflammatory cytokine and defensin production. The expression of *Np5* showed no significant changes (Figure 7I).

**Figure 8:**
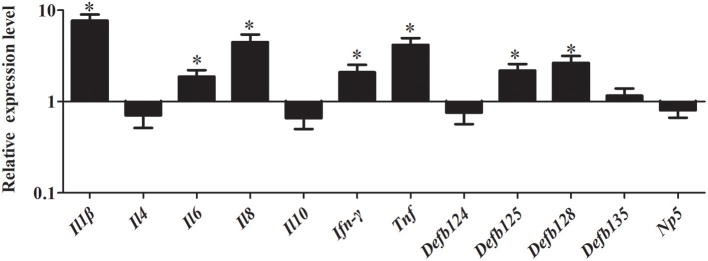
Effect of MDP on expression of immune-related genes induced by EHEC in RK-13 cells. RK-13 cells were treated with 500 ng of MDP for 12 h and then were infected with EHEC. Bars represent the means ± standard deviations (*n* = 3). A significant difference is indicated with an asterisk (*).

As shown in Figure 8, stimulation with MDP significantly induced the expression of immune-related genes in RK-13 cells after EHEC infection. In particular, the pro-inflammatory cytokines, including *Il1*β, *Il8, Ifn-*γ, and *Tnf* were significantly upregulated by 7.69-fold, 4.48-fold, 2.09-fold, and 4.17-fold, respectively (*P* < 0.05). Similarly, the expression of defensins *Defb125* (2.18-fold) and *Defb128* (2.66-fold) was significantly increased.

### Expression of cytokine and defensin induced by rNOD2 after inhibition of the NF-κB

**Figure 9:**
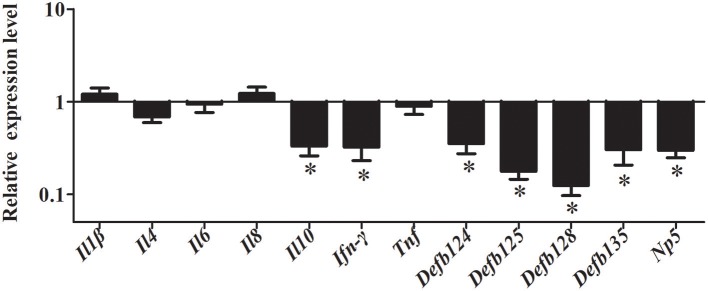
Effect of overexpression of rNOD2 on expression of immune-related genes induced by EHEC after inhibition of NF-κB in RK-13 cells. pC-rNOD2 or pCDNA3.1-empty were transfected into cells for 24 h. RK-13 cells were pretreated with 10 μM of BAY11-7082 for 12 h to inhibit NF-κB pathways and then infected with EHEC. Bars represent the means ± standard deviations (*n* = 3). A significant difference is indicated with an asterisk (*).

To further investigate whether NF-κB pathway was activated by rNOD2, cells were pretreated with inhibitors of NF-κB. After inhibition of the NF-κB pathway, overexpression of rNOD2 unable increase the expression of pro-inflammatory cytokines and defensins induced by EHEC. The expression of all defensins was significantly decreased. Especially, the expression of *Defb125* and *Defb128* was significantly downregulated by 0.18-fold and 0.12-fold (*P* < 0.05). Although the expression of all pro-inflammatory cytokines was reduced, only the expression of *Ifn-*γ was significantly downregulated by 0.32-fold (*P* < 0.05; Figure 9).

### Antibacterial ability

**Figure 10:**
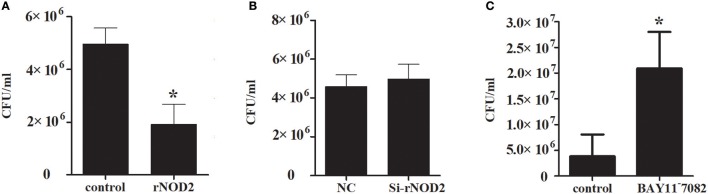
The antimicrobial activity of rNOD2. **(A)** pC-rNOD2 and pCDNA3.1 (+) vector were transfected into RK-13 cells for 24 h. **(B)** Si-rNOD2 and NC siRNA were transfected into RK-13 cells for 24 h. **(C)** After transfection of pC-rNOD2, cells were treated with 10 μM of BAY11^−^7082 for 12 h. The cells were infected with EHEC. The cells were then lysed in 1% Triton X-100, and intracellular bacteria were calculated. Bars represent the means ± standard deviations (*n* = 3). A significant difference is indicated with an asterisk (*).

To explore the ability of rNOD2 to inhibit EHEC, RK-13 cells were infected with EHEC after transfection with rNOD2 and si-rNOD2, and the EHEC counts were calculated. As shown in Figure 10A, the EHEC counts were significantly lower in cells transfected with rNOD2 than those in control cells transfected with pCDNA3.1 (+) vector. In contrast, EHEC counts were not different in cells transfected with si-rNOD2 compared with control cells transfected with NC siRNA (Figure 10B). Moreover, inhibition of the NF-κB pathway eliminated the ability for rNOD2 to inhibit EHEC growth (Figure 10C).

## Discussion

In this report, we cloned rNOD2 in RK-13 cells, and its open reading frame encodes a protein of 1,013 amino acids. Similar to human and mouse NOD2, rNOD2 does not contain signal peptide so that in can release the mature proteins into intracellular space, indicated that rNOD2 is intracellular cytosolic sensors and expressed mainly in the cytosol (Inohara and Nuñez, 2003). A comparison of the deduced amino acid level of rNOD2 with that in *O. princeps, M. nemestrina, Pan troglodytes*, and *Homo sapiens* NOD2 indicated 91.7, 86.5, 86.4, and 86.4% identity, respectively. To understand the evolutionary relationship of rNOD2, phylogenetic tree was generated based on NOD2 protein of various species, and rNOD2 belonged to the branch of mammals. Similar results indicate that rNOD2 was closer related to *O. princeps* NOD2 than to *M. nemestrina, P. troglodytes*, and *H. sapiens* NOD2.

NOD2 is an essential protein for innate immune responses, results of tissue distribution showed a broad expression spectrum. The highest level of rNOD2 was detected in the spleen, duodenum, and jejunum. Similar results have been observed in newborn swine, the NOD2 expressed in varies tissues with different level, and the highest expression in the mesenteric lymph nodes and spleen (Tohno et al., 2008). Previous studies have demonstrated that NOD2 associated with chronic inflammatory disorders of Crohn's disease (Girardin et al., 2003b; Inohara and Nuñez, 2003; Sakiko et al., 2016). NOD2 could recognize gram-negative and gram-positive bacteria in the cytosolic compartment by identification of MDP in pigs (Tohno et al., 2008). To investigated whether rNOD2 was involved in the host antibacterial response in rabbits, EHEC was used to infect the 35-day-old rabbits. In the early stages of EHEC infection, bacteria inhibit the expression of *rNod2* in order to replicate in the spleen. The upregulation of the *rNod2* at 3 dpi indicates the *rNod2* likely participates in the innate immune defense against EHEC infection.

In humans, NOD2 induces the activation of NF-κB, which is mediated through homophilic CARD-CARD interactions with receptor-interacting protein 2 (Ogura et al., 2001). We also investigated the overexpression of rNOD2 in RK-13 cells, and our results revealed that it is able to activate NF-κB luciferase activity, and increase the expression of cytokines and defensins. In particular, the expression of *Il6, Il8, Tnf*, and *Defb135* significantly induced by overexpression of rNOD2. The defensins contribute to innate immunity response and are crucial part of the first line of defense for various pathogenic microbes. They display a broad range of antimicrobial activities and are effective against viruses, gram-negative bacteria, gram-positive bacteria, and fungi (Tavares et al., 2013). Defensins are divided into α-defensins, β-defensins, and θ-defensins in mammals (Wang et al., 2003). Our results concur with studies that NOD2 can mediate antibacterial responses through the NF-κB signaling pathway during inflammatory reaction and increase the expression of pro-inflammatory cytokines as well as antimicrobial peptides in humans (Windheim et al., 2007; Hsu et al., 2008). In another research, overexpression of the effector domains of NOD2 induced the expression of pro-inflammatory cytokines, including *Il1*β, TNF-a, *Il6*, and *Il8*, I- and II-IFN, as well as the antibacterial peptide cathelicidin-2 (Chang et al., 2011). Taken together, rNOD2 plays a major role in activating the NF-κB signaling pathway and promoting the expression of pro-inflammatory cytokines and defensins.

It has been demonstrated that NOD2 is mainly associated with antibacterial immune responses in mammals (Meylan et al., 2006) and is necessary for the activation of NF-κB signaling pathway after interactions with MDP (Barnich et al., 2005). In the current study, overexpression of rNOD2 increased EHEC-stimulated NF-κB activities, whereas rNOD2 specific siRNA reduced it. These results demonstrate that rNOD2 recognizes EHEC, resulting in the activation of NF-κB. A previous study has demonstrated that the activation of NOD2 strengthens epithelial innate immunity via increase the production of antimicrobial peptide human beta-defensin-2 (Voss et al., 2006). As expected, our results revealed that the overexpression of rNOD2 may also increase the EHEC infection-induced pro-inflammatory cytokines, including *Il1*β, *Il6, Ifn-*γ, and *Tnf*, as well as β-defensins, including *Defb124, Defb125*, and *Defb128*. In contrast, knockdown of rNOD2 inhibited the expression of pro-inflammatory cytokines and β-defensins. Our results are consistent with a previous research that macrophages from NOD2^−/−^ mice could not produce pro-inflammatory cytokines or undergo NF-κB activation after MDP challenge (Kobayashi et al., 2005). In addition, stimulation of rNOD2 with MDP significantly induced the expression of pro-inflammatory cytokines and defensins in EHEC-infected RK-13 cells. The NOD2 ligand, MDP can be added to the new EHEC vaccine to promote the innate immune response. In the current study, the overexpression of rNOD2 inhibited the growth of EHEC in RK-13 cells, while knockdown of rNOD2 showed no effect on the replication of EHEC compared to that of control. Importantly, in the context of NF-κB signaling inhibition, rNOD2 was unable to inhibit the growth of EHEC and downregulated the expression of pro-inflammatory cytokines and defensins. These results deduce that the antibacterial activity and induction of immune-related genes for rNOD2 is mediated through NF-κB signaling. rNOD2 contributed to the inhibition of intracellular bacterial growth, which are in turn involved in the initiation of inflammation and an antibacterial response (Smith et al., 2000).

In conclusion, we cloned the rNOD2 gene from RK-13 cells and demonstrated that it was widely distributed in the tissues of rabbits. rNOD2 was involved in immune response to the EHEC infection *in vivo*. In addition, rNOD2 might activate NF-κB signaling and promote the expression of pro-inflammatory cytokine and defensins in RK-13 cells infected with EHEC. Most important is that rNOD2 can inhibit the growth of EHEC by activating NF-κB *in vitro*. In the future, it is believed that rNOD2 will be provided novel therapeutic perspective against bacterial infections based on its antibacterial ability.

## Author contributions

MG and RL conducted the study and wrote the manuscript. QX and XF collected samples. NL and YS analyzed data. LW and TC designed the study and revised the manuscript.

### Conflict of interest statement

The authors declare that the research was conducted in the absence of any commercial or financial relationships that could be construed as a potential conflict of interest. The reviewer SM and handling Editor declared their shared affiliation.
